# Effect of long-term application of bioorganic fertilizer on the soil property and bacteria in rice paddy

**DOI:** 10.1186/s13568-023-01559-2

**Published:** 2023-06-13

**Authors:** Zu-ren Li, Si-quan Luo, Ya-jun Peng, Chen-zhong Jin, Du-cai Liu

**Affiliations:** 1 Key laboratory of Pesticide Assessment, Ministry of Agriculture and Rural Affairs, Beijing, P.R. China; 2 Hunan Provincial Key Laboratory for Biology and Control of Weeds, Hunan Academy of Agricultural Sciences, Changsha, 410125 China

**Keywords:** Bioorganic weeding fertilizer, Long-term, Rice paddy, Bacterial community, Soil properties

## Abstract

The application of novel bioorganic fertilizer (BIO) has been established as a weed biocontrol strategy, and reduce herbicides pollution and negatively effects on agricultural ecosystems. However, its long-term influences on soil bacterial communities are unknown. Here, 16 S rRNA sequencing to identify the changes that occur in soil bacterial community and enzyme under BIO treatments after five years in a field experiment. BIO application effectively controlled weeds, however no obvious differences between treatments were observed under BIO-50, BIO-100, BIO-200 and BIO-400 treatment. *Anaeromyxobacter* and *Clostridium_ sensu_ stricto_1* were the two dominant genera among BIO-treated soil samples. The BIO-800 treatment had a slight influence on the species diversity index, which was more remarkable after five years. The seven significantly-different genera between BIO-800 treatment and untreated soils included *C._sensu_stricto_1*, *Syntrophorhabdus*, *Candidatus_Koribacter*, *Rhodanobacter*, *Bryobacter*, *Haliangium*, *Anaeromyxobacter*. In addition, BIO application had different effects on soil enzymatic activities and chemical properties. The extractable P and pH saliency correlated with *Haliangium* and *C._Koribacter*, and *C._sensu_stricto_1* observably correlated with exchangeable K, hydrolytic N and organic matter. Taken together, our data suggest that BIO application effectively controlled weeds and a slight influence on soil bacterial communities and enzymes. These findings expand our knowledge of the application of BIO as widely used as a sustainable weed control in rice paddy.

**Supplementary Information:**

The online version contains supplementary material available at 10.1186/s13568-023-01559-2.

## Introduction

Weeds have negative effects on the germination and yield of crop plants. The application of synthetic herbicides is by far the most common method of weed control. However, the excessive use of weed killers pollutes the environment and negatively affects agricultural ecosystems. Currently, the biocontrol of weeds has shown to be a practical weed management tool with ecological benefits. For example, *Cassytha pubescens*, a parasitic shrub, showed to be an effective biocontrol tool when it was used on smaller hosts (Cirocco et al. 2020). Although biocontrol strategies have shown promising results in wide applications, more field-based studies on their influence on non-target organisms are well-considered (Sutton et al. 2021). Less than 1% of direct non-target attack was recorded in an experiment where the impacted plant species had been tested pre-release and was deemed not at risk (Hinz et al. 2020). Hence, the influence of the biocontrol of weed on agricultural ecosystems require more evaluations.

Soil bacteria serve as early indicators of changes in agricultural ecosystems (induced by natural and anthropogenic disturbances), as they have shown to be sensitive to physical and chemical variations from weed management (Gupta & Singh 2018; Cagnini et al. 2019). Numerous studies have reported on the responses of soil bacteria to herbicide applications. For example, the application of 35 g/hm^2^ and 70 g/hm^2^ of bispyribac sodium (to inhibit rice weeds), had an impact on the soil microbial population, enzyme activities and functional microbial diversity in paddy soil (Kumar et al. 2020). Also, the application of 500 µmol of 4-chloro-2-methylphenoxy acetic acid, significantly reduced the relative abundance of Cyanobacteria-chloroplasts (Zhang et al. 2021). However, another study showed that the application of herbicides did not have a significant impact on soil microbial community. The application of 130 mg/kg of halosuluron methyl did not disturb the soil bacterial community (Wang et al. 2020). Thus, soil bacteria are one of indicators of eco-friendly agricultural practices in farmland ecosystems.

In our previous study, a novel bioorganic weeding fertilizer (BIO) have been obtained by fermenting mature compost with kitchen garbage, maize straw, wood-destroying fungal dregs, rice straw, tobacco straw, plant ash, chicken, and sheep manure. The novel BIO was found to be effective in controlling grass and broad-leaved weeds in three rice fields (Huanan, Hainan, and Heilongjang, in China) for two years (2014 and 2015) with an average weed suppression rate of more than 80% (Li et al. 2018). The application of BIO did not disturb the main community structure and functions of soil bacteria in multi-site field experiments (Li et al. 2021). However, the BIO effects on soil bacteria in rice paddy in the long-term are not well-known. As the previous report, the soil ammoniaoxidizing bacteria was more diverse in the long-term application of mineral fertilizer (Chu et al. 2007). The farmyard manure long-term application could significantly increased the community densities of cellulolytic bacteria in Phaeozem (Ulrich et al. 2008). Hence, the long-term effects on soil bacteria is necessary to broad-used of BIO in the rice paddy.

Is or is not the long-term application of BIO in rice paddy influence on soil bacteria? In the present study, we analyzed the weed-control effect of BIO in rice paddies after five years of trials. We also evaluated BIO-affected soil chemical properties, enzymatic activities and bacterial community composition by 16 S rRNA- sequencing. These results may be answer the question of long-term effect on soil.

## Materials and methods

### Bio-organic fertilizer (BIO) manufacturing

The organic substrates in the BIO were composed of kitchen garbage, maize straw, wood- destroying fungal dregs, rice straw, tobacco straw, plant ash, and chicken and sheep manure. The physical and chemical properties of the compost material measured were provided in our previous study (Li et al. 2018, 2021). The combined process of ZF-5.5 mechanical fertilizer preparation and pile fermentation was used to produce composting manure at a temperature range of 40-80 °C for 15 days. Man-made heating and cooling were used to control temperature on the first day. The compost was moved out and piled fermentation began one day later. After 15 days, the compost turned taupe gray, exhibited threadiness and had a slightly sour fragrance. This compost contained 53.4% organic matter, 2.0% N, 3.7% P_2_O_5_, and 1.1% K_2_O.

### Field experiment and soil sample collection

Our study site was a field located in Gaoqiao, Changsha, Hunan Province, China (N28°28ʹ20″, E113°4ʹ51″), which had been cropped rice-rice per year (from April to October, mean annual precipitation and temperature in the last three years were 1427.41 mm and 18 °C) and had already been carried out for 30 years. The field trial started with the preparation of eight 40 m×20 m plots on April 26, 2017. The rice plants (variety Longxiang 32) were transplanted to a density of 25 plants/m^2^. Three days after transplantation (on April 29), BIO and common fertilizer (CBF, contained 54.4% organic matter, 1.8% N, 3.5% P_2_O_5_, and 1.2% K_2_O, Changsha Beye Agricultural Ltd., Changsha, China) were spread over the plots. Twenty-four treatments were set up in the experiment, including BIO (750 kg/hm^2^ (BIO-50), 1500 kg/hm^2^ (BIO-100), 3000 kg/hm^2^ (BIO-200), 6000 kg/hm^2^ (BIO-400), and 12,000 kg/hm^2^ (BIO-800); 25 g/L of herbicide Penoxsulam OD (HP, Dow Agro Sciences); 1500 kg/hm^2^ CBF; hand weeding; and an untreated control (CK, without weed management strategy). Each individual plot (1 m × 1 m) was separated by ridges to prevent water channeling, with three replicates in a randomized block arrangement. All field management practices were in line with local practices, except for the irrigation during BIO application, as a 3–5 cm water layer had to be maintained for 7 days. After this period, irrigation was conducted traditionally. The application of base fertilizer was decreased to 25% of normal dosage (CBF, 6750 kg/hm^2^). No top-dressing and other weed management practices were carried out in these plots. The second group of rice plants were transplanted in July and then harvested in October. The same experimental treatments were carried out in the plots. The traditional rice agronomical management strategies, as described by Zou YB (1999), were used. The first batch of soil samples were collected from all plots on May 27th, 2017, at one month after BIO application. One hundred grams (100 g) of surface soil (0–15 cm) was collected from 30 points in each plot and then every 3 points were mixed thoroughly together in plastic bags as 10 samples from each treatment. Each soil samples were sieved at 2 mm and then randomly divided into two parts, one part was frozen and stored at -80 °C, and the other part was air dried for one week and stored at 25 °C. For the next four years (2018, 2019, 2020, 2021), the plots were manually tilled without much disturbance. The same experimental treatments were carried out in the plots. Farm operations were consistent over the five years. The second batch of soil samples were collected from all plots on June 1st, 2021.

### Weed control effect assay

The effects of BIO application on weeds in the fields were evaluated on May 27th, 2017 and June 1st, 2021. Three points (1 m^2^) were randomly chosen in each plot and the number of grass and broad-leaf weed species were recorded separately. Aboveground fresh weed biomass was measured at 30 days after BIO application. Control effect (%)= (CK-Tt)/ CK× 100; CK: number or fresh weight of untreated control plots weeds, Tt: number or fresh weight of BIO, CBF and HP plots weeds plants.

### DNA extraction and MiSeq sequencing

Sample soil DNA was extracted using the MoBioPower Soil DNA Isolation Kit (MO BIO, San Diego) following the manufacturer’s protocol. After quantification using Nanodrop (ND-1000 Spectrophotometer; Nanodrop Products, Wilmington, USA), the V4 hyper variableregion of the 16 S rRNA gene was amplified with the following primer pair: 515 F (5′-GTGCCAGCMGCCGCGGTAA-3′) and 806R (5′-GGACTACHVGGGTWTCTAAT-3′). Amplicon quality was visualized by agarose gel electrophoresis. The amplicons were purified using the AMPure XP beads (Agencourt), and then amplified in a second round of PCR. The PCR process was caried out as a 20 µL mixture containing 5 µL FastPfu buffer, 2 µL dNTPs, 1 µL primer, 0.5 µL FastPfu Polymerase, 2µL DNA and 8µL water. Following another round of purification using the AMPure XP beads, the final amplicons were quantified using the Qubit dsDNA assay kit. Equal amounts of purified amplicons (200 ng) were pooled for library construction and subsequent PE125 sequencing using the Illumina MiSeq platform (Illumina, San Diego, CA) with the MiSeq 500 cycles kit.

### Sequence pre-processing and statistical analysis

Sequence processing was conducted using the Galaxy pipeline (http://zhoulab5.rccc.ou.edu: 8080/ root) following a previous study (Bolger et al. 2014). Briefly, the raw sequences were assigned to samples by “Detect barcodes” script, and ambiguous bases (N) were detected and cut off using the Trimmomatic software (Wang et al. 2007). The cut off values for low-quality sequences were set at an average quality score of 20, and these sequences were eliminated using the sliding window trimming approach. Forward and reverse reads, with at least 10-bp overlap and less than 5% mismatch, were then combined using Flash. The shorter sequences and chimeras were removed from the combined sequences using the QIIME software (version 1.8.0). Operational taxonomic units (OTUs) clustering was performed using UCLUST at 97% similarity level, and taxonomic assignment was conducted using the Ribosomal Database Project (RDP) classifier, with a minimal of 50% confidence estimate. Samples were rarefied at 19,600 sequences per sample, and these were classified into 15,706 OTUs. All data were translated into OTU relative abundance table for subsequent analysis.

Alpha diversity indices of the microbial community, including Shannon-Weiner’s and Chao1 indices, were calculated using the “Vegan” package. The Chao1 diversity index was calculated as reported in a previous study (Qin et al. 2019). Beta diversity was analyzed using the principal coordinates analysis (PCA), carried out using the “Vegan” package based on shared branches of weighted unique fraction (UniFrac) distances. Redundancy analysis (RDA) was performed using the R Vegan package to determine the nonlinear relationships between the soil chemical properties and microbial properties. Co-occurrence networks of significantly bacterial communities as according the previous report (Lasa et al. 2022). Test of differences among the weed control efficiency and soil chemical properties data (Three replicates) were performed with ANOVA and subsequently with LSD test using the SSPS software. Test results with a *p* value < 0.05 were considered statistically significant.

### Soil chemical properties and enzymatic activity measurement

Soil sample pH was measured in soil-water solution (W/V 1:5). Total N and K content was measured using an elemental analyzer (Carlo Erba, Milan, Italy) and total P content was measured calorimetrically using the molybdate method. Hydrolytic N, extractable P, exchangeable K, and organic matter content were measured as described previously (Tao et al. 2018). Soil representative enzymes activities of the second soil sample were measured following instructions given by the kits used (Solerbio life Sciences, Beijing, China). Soil urease (S-UE) was defined as 1 g of soil which produced 1 µg “NH3-N” (U/g) daily. Soil acid phosphatase (S-ACP) was determined as 1 g of soil which liberated 1 nmol phenol at 37℃ (U/g) daily. Soil β-glucosidase (S-β-GC) was determined as 1 g of soil which produced 1 µmol p-nitroohenol (U/g) daily (Wade et al. 2021). All soil chemical properties and enzyme assays were conducted in duplicate.

## Results

### BIO application effectively controlled weeds in rice paddy

**Fig. 1:**
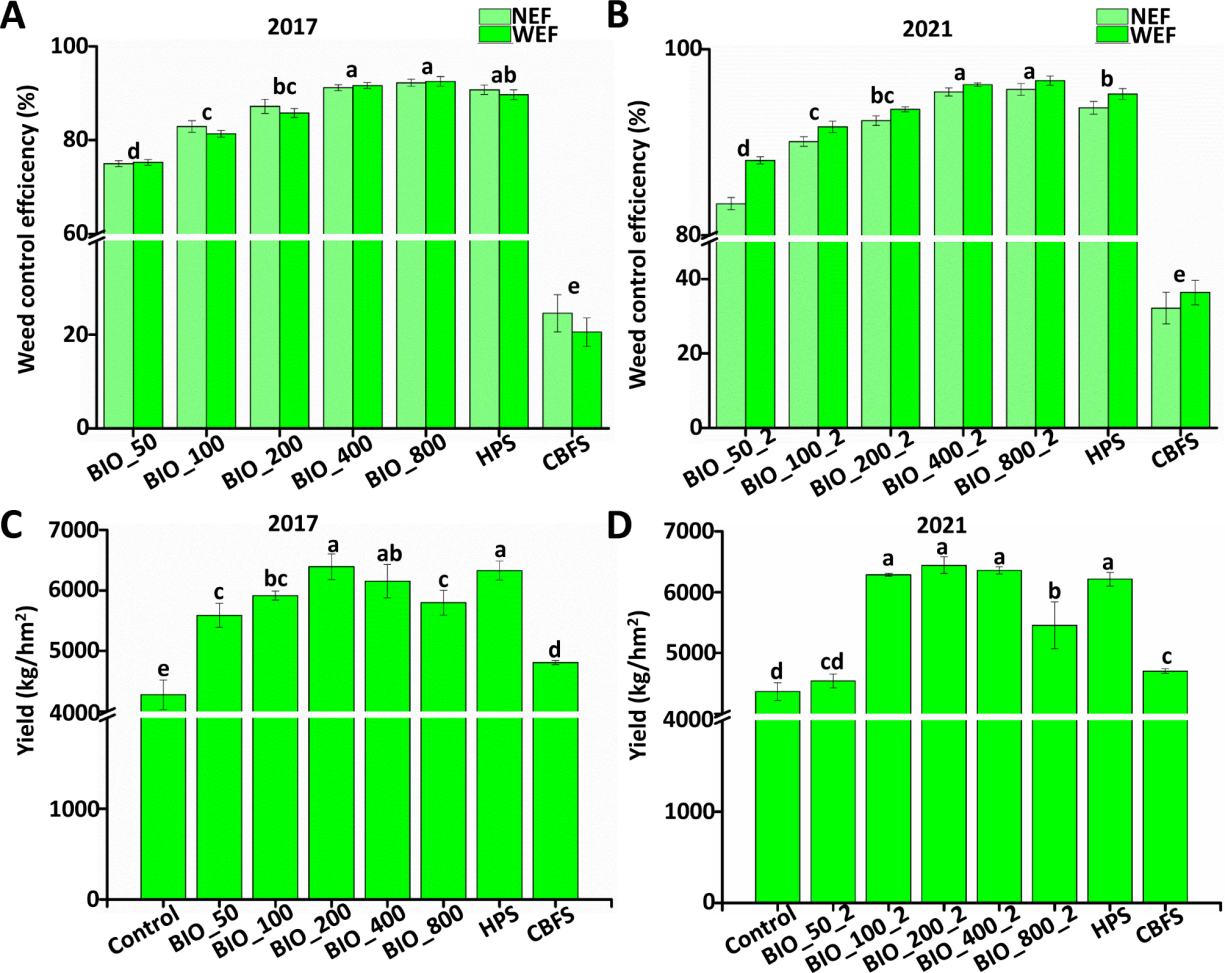
Effect on weed and rice yield after long-term application of BIO. **A**: BIO dosage effect on weed control in 2017; **B**: BIO dosage effect on weed control in 2021. **C**: BIO dosage effect on rice yield in 2017; **D**: BIO dosage effect on rice yield in 2021. BIO-50: 750 kg/hm^2^ BIO-treated; BIO-100: 1500 kg/hm^2^ BIO-treated; BIO-200: 3000 kg/hm^2^ BIO-treated; BIO-400: 6000 kg/hm^2^ BIO-treated; BIO-800: 12,000 kg/hm^2^ BIO-treated;NEF: weed number control effect (%) (plants/m^2^); WEF: weed fresh weight control effect (%) (g/m^2^). Data analysis is based on the average of nine repetitions. Means with the same letter are not significantly different according to Fisher’s protected LSD test (a = 0.05)

The weed community in the treatment plots comprised both grass weeds (*Echinochloa crus-galli*, *Cyperus iria*, *Leptochloa chinensis*, and *Scirpus planiculmis*) and broadleaved weeds (*Ludwigia prostrata*, *Monochoria vaginalis*, *Lindernia procumbens*, *Eleocharis yokoscensis*, *Ammannia baccifera*, and *Potamogeton distinctus*). In 2017, application of BIO had good effects in controlling *E. crus-galli*, *L. prostrata*, and the total weeds (Fig. 1A). Thirty days after fertilization, the control effect (EF) of BIO-50 treatment on the total weed number (N) was 75%, whereas that of BIO-100, BIO-200, BIO-400, and BIO-800 treatments was above 80%, except for BIO-100 on *E. crus-galli*. The control effect on the fresh weight was 75.20% for the BIO-50 treatment, and above 80% for the BIO-100, BIO-200, BIO-400, and BIO-800 treatments. With an increase in BIO dosage, the control effect on weed plants increased, but these differences were not significant. Under the BIO-200 treatment, the weed control effect was equivalent to that of the herbicide (25 g/L Penoxsulam OD). Interestingly, the control efficiency of all treatment was above 80% in 2021, including that of BIO-50 which was 83.43% (Fig. 1B). An increase in BIO dosage cause an increase in control effect, but these differences were not significant.

Field experiment showed that the application of bio-organic fertilizer had positive effects on rice yield. After fertilization, rice yield under the BIO-50 treatment was 5587.05 kg/hm^2^, an increase of above 42.67% compared to the control. Under the BIO-200 treatment, the rice yield was similar to that under herbicide treatment (25 g/L Penoxsulam OD). An increase in BIO dosage (form BIO-50 to BIO-400), resulted in an increased in rice yield in 2017, but these differences were not significant (Fig. 1C). However, the rice yield decreased after application of BIO-800 compared to BIO-400. In 2021, the rice yield increased after application of BIO from BIO-50 to BIO-400 (Fig. 1D). The above results indicated that BIO application was an effective weed management strategy, that also improved rice yield.

### BIO application had no significant influence on soil bacterial communities after the first year

**Fig. 2:**
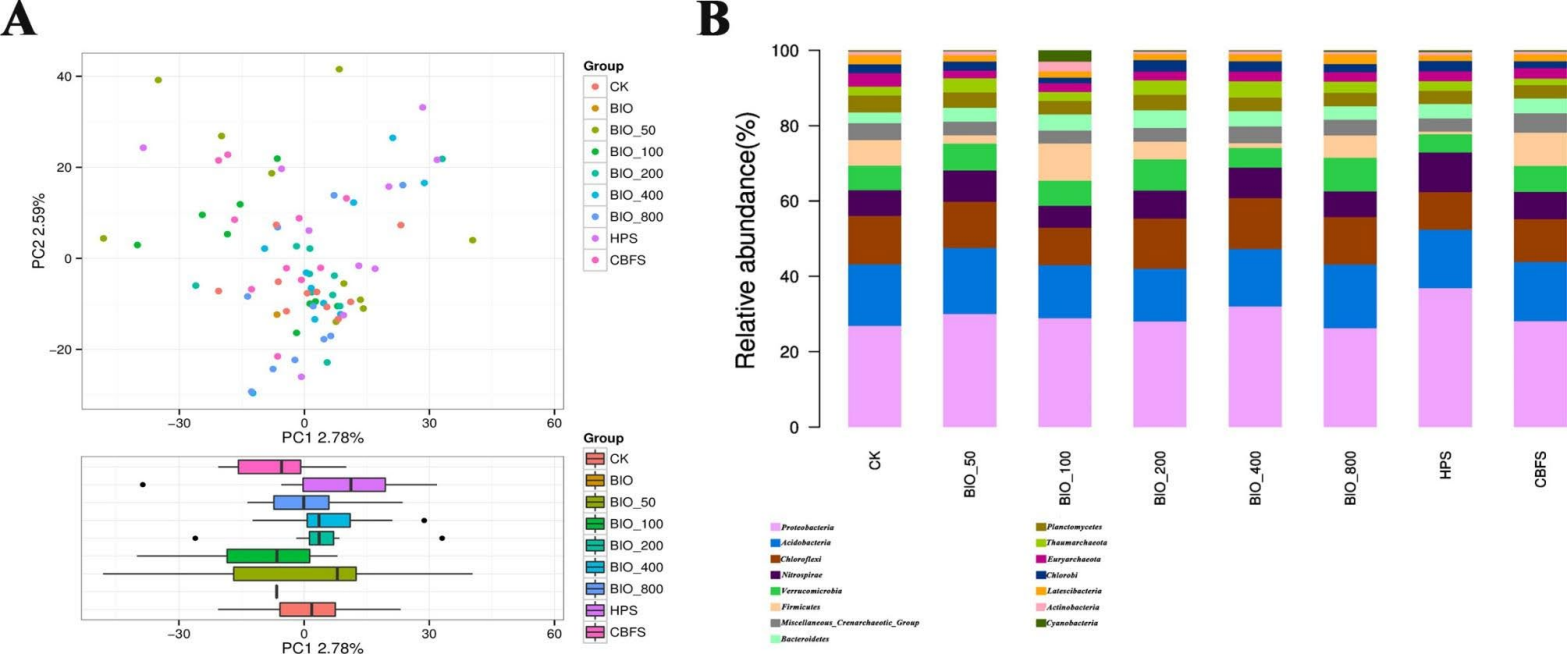
BIO application had no significant influence on soil bacterial communities in 2017. **A**: Beta-diversity indices of bacterial community structure in BIO-treated soil samples, CBF soil samples, HP soil samples, CBF samples, and BIO samples (PCA plot); **B**: Bacterial diversity, as represented by the relative abundances (%) of different phylum in BIO-treated, CBF, and HP soil samples, and CBF and BIO samples, respectively. “others” refers to 16 S sequence analysis that are not strictly associated with phylum; BIO: BIO sample; BIO_50: 750 kg/hm^2^ BIO-treated soil in 2017; BIO_100: 1500 kg/hm^2^ BIO-treated soil in 2017; BIO_200: 3000 kg/hm^2^ BIO-treated soil in 2017; BIO_400: 6000 kg/hm^2^ BIO-treated soil in 2017; BIO_800: 12,000 kg/hm^2^ BIO-treated soil in 2017; HPS: herbicide-treated soil in 2017; CBF: common bio-fertilizer in 2017; CBFS: common bio-fertilizer-treated soil in 2017; and CK: untreated control soil in 2017

**Fig. 3:**
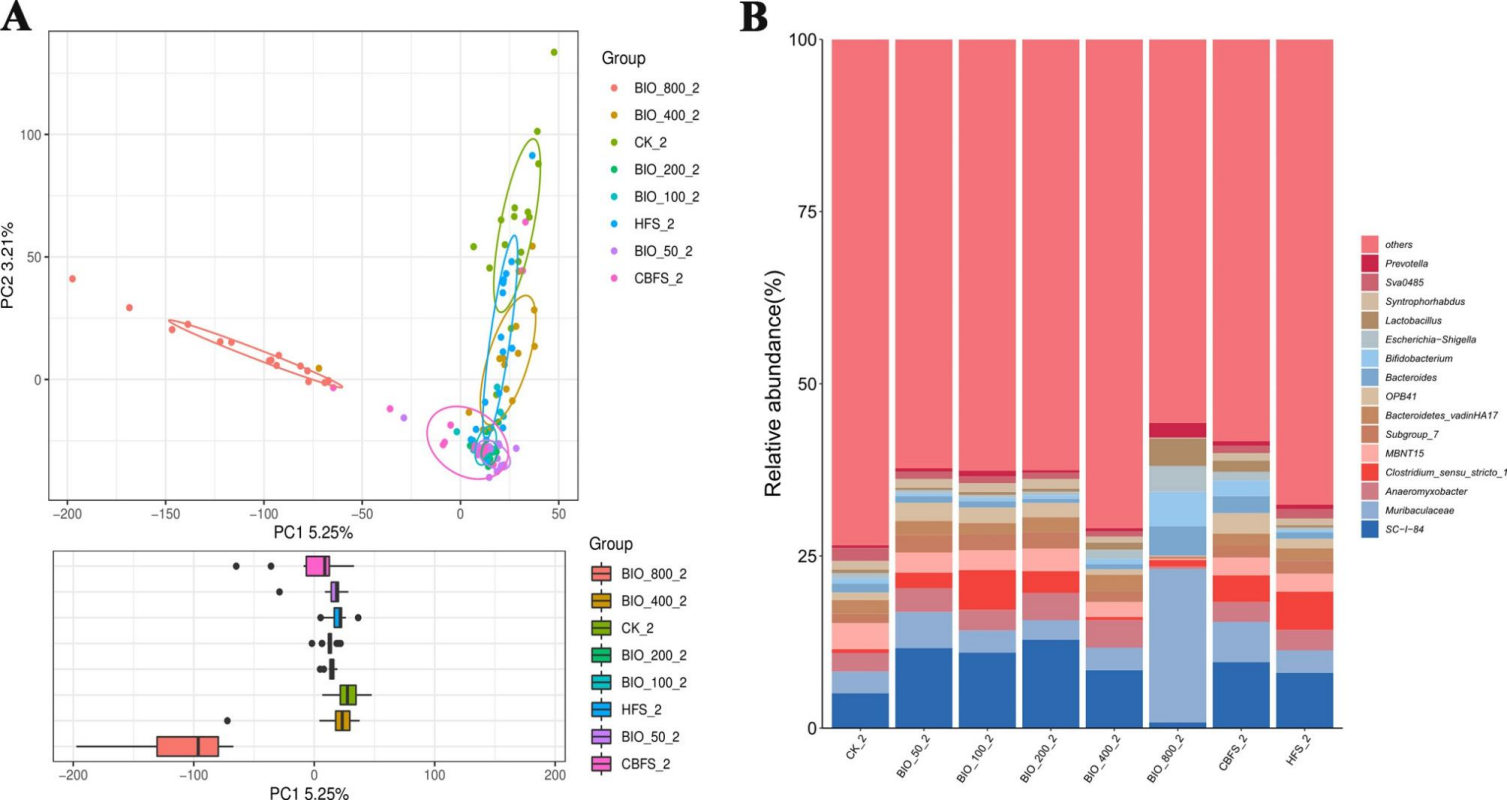
Effect of BIO application on soil bacterial communities in 2021. **A**: Beta-diversity indices of bacterial community structure in BIO-treated soil samples, CBF soil samples, HP soil samples, CBF samples, and BIO samples (PCA plot); **B**: Bacterial diversity, as represented by the relative abundances (%) of different genera in BIO-treated, CBF, and HP soil samples, and CBF and BIO samples, respectively. “others” refers to 16 S sequence analysis that are not strictly associated with genus. BIO: BIO sample; BIO_50_2: 750 kg/hm^2^ BIO-treated soil in 2021; BIO_100_2: 1500 kg/hm^2^ BIO-treated soil in 2021;BIO_200_2: 3000 kg/hm^2^ BIO-treated soil in 2021; BIO_400_2: 6000 kg/hm^2^ BIO-treated soil in 2021; BIO_800_2: 12,000 kg/hm^2^ BIO-treated soil in 2021; HPS_2: herbicide- treated soil in 2021; CBF_2: common bio-fertilizer in 2021; CBFS_2: common bio-fertilizer-treated soil in 2021, and CK_2: untreated control soil in 2021

**Fig. 4:**
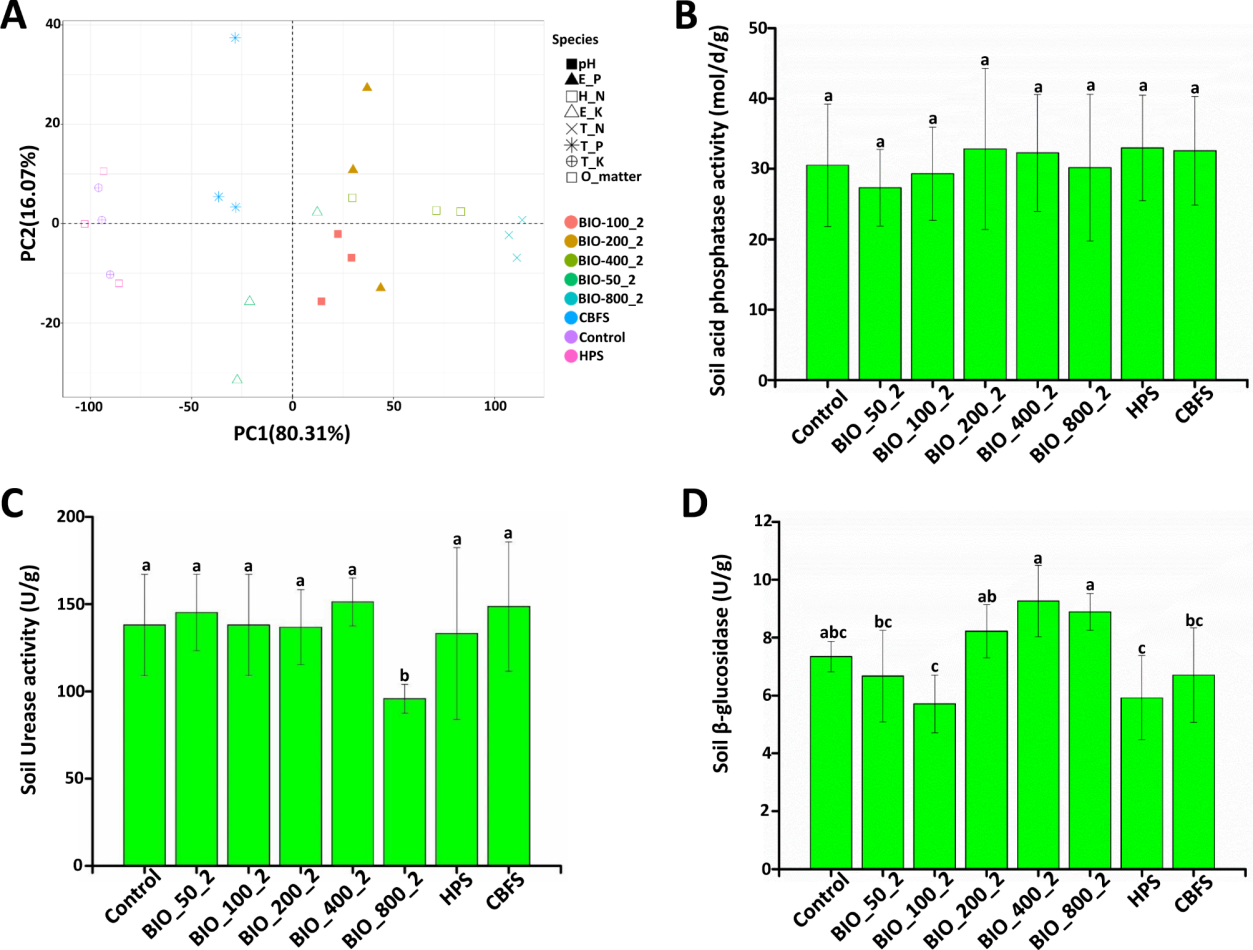
Effect on soil chemical properties and enzymes after BIO application in 2021. **A**: PCA analysis of chemical properties of the surface soil layer (0-15 cm) from the BIO-treated; **B**: The activity of soil acid phosphatase activity (S-ACP) was not influenced by BIO-treatment compared to CK; **C**: BIO influence on soil urease activity (S-UE); **D**: the soil β-glucosidase (S-β-GC) activity was obviously influenced under BIO-treatment. Values shown here represent the average of three repetitions (n = 3). Means with different letters represent significant differences at p < 0.05. BIO_50_2: 750 kg/hm^2^ BIO-treated soil in 2021; BIO_100_2: 1500 kg/hm^2^ BIO-treated soil in 2021;BIO_200_2: 3000 kg/hm^2^ BIO-treated soil in 2021; BIO_400_2: 6000 kg/hm^2^ BIO-treated soil in 2021; BIO_800_2: 12,000 kg/hm^2^ BIO-treated soil in 2021; HPS: herbicide- treated soil in 2021; CBF: common bio-fertilizer in 2021; CBFS: common bio-fertilizer-treated soil in 2021, and CK: untreated control soil in 2021

Sequencing the V1-V9 region of 16 S rRNA genes revealed a diverse bacterial community composition and dynamics. The number of OTUs in all samples were 405–1633, and 47 OTUs were common in all 2017 samples, as shown by the flower plot, which belonged to 60 phyla, 437 families and 625 genera (Fig. 1S). The alpha-diversity analysis (Shannon and chao1) of the different soil treatments (BIO, CBF and HP) are shown in Figs. 2S and 3S. Compared the untreated control (CK), most of BIO_treated soil samples displayed uniform species abundance indices, but the dosage of BIO (BIO_800) had a slight effect on the diversity index. Furthermore, there were no significant differences among the CBF, BIO, and HP soil samples (P > 0.05). For beta-diversity analysis of soil microbial community, PCA of the OTU was carried out PCA plot indicated that all replicates of treated soils (CBF, HP, CK, and low dose of BIO) clustered together (Fig. 2A). The first and second axes explained 2.78% and 2.59% of the variance, which totaled 5.37% of the cumulative variance. But the dose of BIO (BIO_800) had a slight effect on the diversity index, which revealed that the soil community under this treatment was different from the other treatments. The soil bacterial community was not significantly different among the CBF, BIO, and HP soil samples, except for BIO_800 samples. Bacterial community structures (at the phylum level) in the BIO-treated, CBF, and HP soil samples are shown in Fig. 2B. The five most dominant phylum among the BIO-treated soil samples were *Proteobacteria*, *Acidobacteria*, *Chloroflexi*, *Nitrospirae*, and *Verrucomicrobia.* Compared with CK, there was no obvious difference among all BIO-treatments. The relative abundance of *Proteobacteria* ranged from 19 to 42.82% under BIO_50 treatment (average 27.81%), 12.21–47.98% under BIO_800 treatment (average 23.81%), and 16.03–37.41% in CK (average 23.5%). The five most dominant genera among the BIO-treated soil samples were *Anaeromyxobacter*, *Candidatus Nitrosotalea*, *Clostridium sensustricto1*, *Haliangium*, *Candidatus*, and *Nitrotoga* (Fig. 4S). Similar results were exhibited by the five dominant phyla. Hence, bacterial community structures was not significantly different in the BIO-treated, CBF, and HP soil samples after the first year.

### BIO application had influence on soil bacterial communities in 2021

**Fig. 5:**
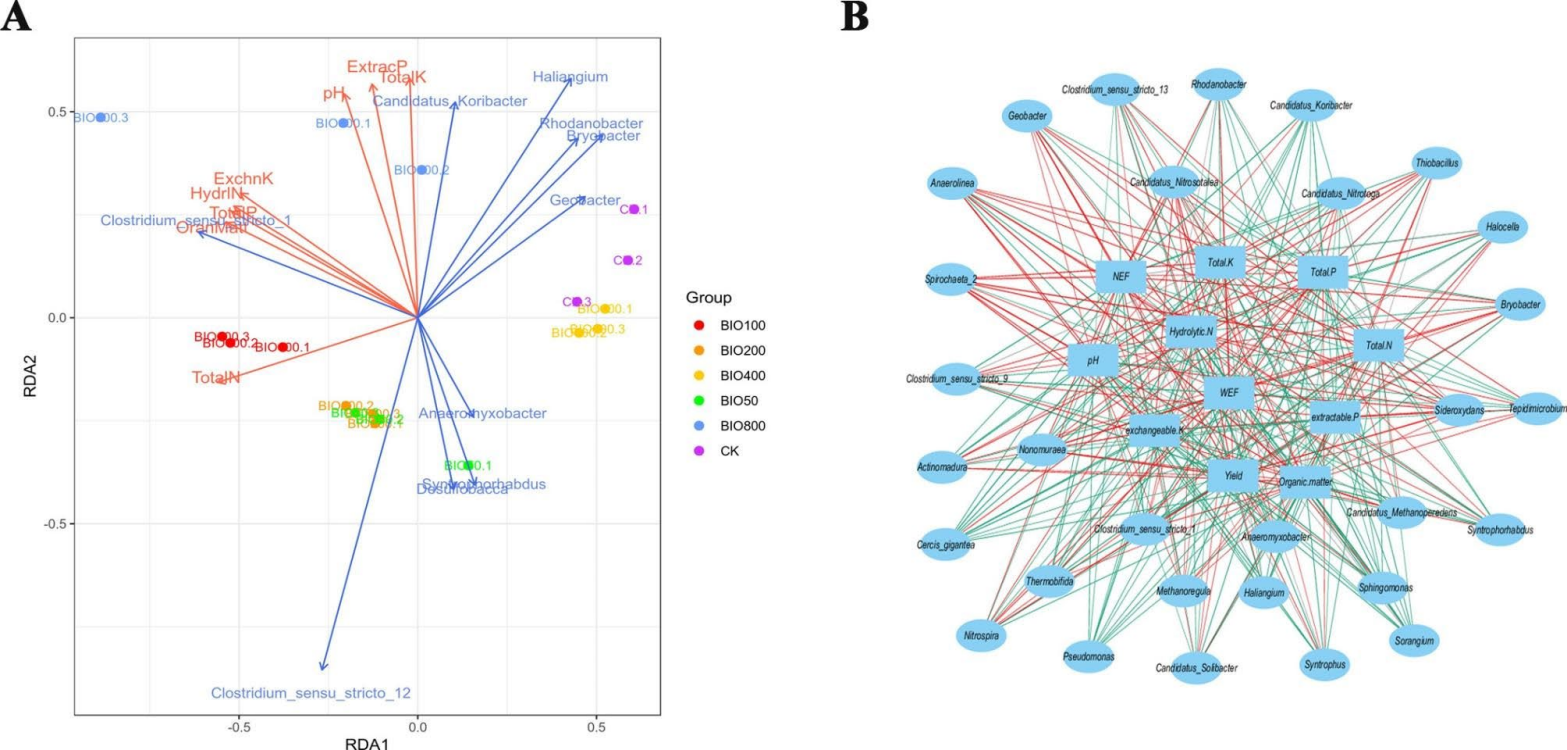
Correlation analysis of BIO effect on soil different bacterial composition and chemical properties. **A**: Redundancy analysis (RDA) of soil differentially active soil bacterial genera and chemical properties (*p* value = 0.02). **B**: The correlation analysis of yield, weed control efficiency, soil chemical properties and bacteria communities. Sample names denote the following: BIO50: 750 kg/hm^2^ BIO-treated soil in 2017 and 2021; BIO100: 1500 kg/hm^2^ BIO-treated soil in 2017 and 2021; BIO200: 3000 kg/hm^2^ BIO-treated soil in 2017 and 2021; BIO400: 6000 kg/hm^2^ BIO-treated soil in 2017 and 2021; BIO800: 12,000 kg/hm^2^ BIO- treated soil in 2017 and 2021; HPS: herbicide-treated soil in 2017 and 2021; CBF: common bio-fertilizer in 2017 and 2021; CBFS: common bio-fertilizer-treated soil in 2017 and 2021, and CK: untreated control soil in 2017 and 2021

To evaluate the long-term effect on the soil bacterial communities after BIO application, we also sequenced the 16 S rRNA genes of BIO-treated samples in 2021. The number of OTUs was 3757–6984 in all samples, and there were differences among 11,980 OTUs, which belonged to 61 phyla, 669 families and 1495 genera (Fig. 5S). The alpha diversity analysis of different soil treatments (BIO, CBF and HP) was represented by the Chao1 and Simpson violin. Results of the chao1 analysis indicated that application of BIO had an effect on the degree of diversity of soil bacterial communities (Fig. 6S). The chao1 values recorded under BIO_400_2 and BIO_ 800_2 were lower compared to the CK. Similarly, the Simpson indices displayed uniform species abundance among BIO-treated soil samples, and the highest dose of BIO had effect on the diversity index (Fig. 7S). PCA analysis of beta diversity indicated that all replicates of treated soils (CBF, HP, CK, and BIO) clustered together except for the BIO_800_2 samples (Fig. 3A). The first and second axes explained 5.25% and 3.21% of the variance. The BIO_800_2 samples were away from other samples in the PCA axes. Bacterial community structures (at the genus level) in the BIO-treated, CBF, and HP soil samples are shown in Fig. 3B. The five most representative genera among the BIO-treated soil samples were *SC-I-84*, *Muribaculaceae*, *Anaeromyxobacter*, *Clostridium sensu stricto1*, and *MBNT15*. Compared with CK, there was no obvious difference among all BIO-treatments exclude BIO_800_2. In the BIO_800_2 treatment, the five most genera were still *SC-I-84*, *Muribaculaceae*, *Anaeromyxobacter*, *Clostridium sensu stricto1*, and *MBNT15*, but the relative abundances were significantly changed. The relative abundance of *Clostridium sensu stricto1* ranged from 0.03 to 3.69% under BIO_50_2 treatment (average 2.27%), 0.35–8.05% under BIO_800_2 treatment (average 0.95%), and 0.15–1.21% in untreated control (average 0.56%). Hence, BIO-treated soils had influence on the bacterial community structure five years after the first year assessment.

### Application of BIO had influence on soil chemical properties and soil enzymes

BIO application had different effects on soil chemical properties (Tables 1S and 2 S). In 2017, there was no significant difference in the Extractable P content (values ranged from 0.21 to 0.45 mg/kg in all samples) between BIO treatments and untreated control (CK) samples, and between the HPS and CBFS treatments. Soil pH values under the BIO_50_2 and BIO_100_2 treatments were nearly the same as that of the untreated control, but an increased dosage of BIO (BIO_200_2 to BIO_800_2) increased these values by 3.36-4.46% compared to the untreated soil. There were marginal differences in the recorded pH values among HPS, CBFS, and CK. Similar results were obtained for the total K, total N, total P, Exchangeable K, Hydrolytic N, and Organic Matter. Interestingly, the PCA analysis results show that some chemical properties such as pH, total K, total N and total P still did not change after BIO long-term application. However, BIO had significant effects on other chemical properties in the long-term (Fig. 4A). The Exchangeable K, Extractable P, Hydrolytic N, and Organic Matter in BIO_800_2 treatment increased by about 167.62%, 769.16%, 61.56%, 30.56% respectively compared to CK. The other dosages of BIO application also had influence on these chemical properties. These results demonstrate that application of BIO in paddy fields changed the soil chemical properties from 2017 to 2021.

Similarly, BIO application had different effects on soil enzymatic activities. The S-ACP activity was not influenced by BIO-treatment compared to CK in 2021 (Fig. 4B). The S-UE activity was also not significantly affected by BIO-treatment except under BIO-800 treatment. Under BIO-800 treatment, S-UE activity decreased by 43.19% compared to CK (Fig. 4C). The S-β-GC activity was obviously influenced under BIO-treatment; it decreased under BIO_100_2 and BIO_200_2 treatments compared to CK, but increased under BIO_400_2 and BIO_800_2 treatments (Fig. 4D). The above results show that BIO application had different levels of effects on soil chemical properties and soil enzymes.

### Correlation analysis of BIO-800 effects on soil different bacterial composition

To understand how the application of BIO-800 influenced soil bacterial communities, compared to the control, we further carried out a combined analysis of the significant differences among the genera between 2017 and 2021. The 7 genera found to show differences under BIO-800 treatment included *Clostridium_sensu_ stricto_1*, *Syntrophorhabdus*, *Candidatus_Koribacter*, *Rhodanobacter*, *Bryobacter*, *Haliangium*, and *Anaeromyxobacter* (Fig. 8S). Of these, *Clostridium_ sensu_stricto_1* showed the most negative association with BIO treatment among the 7 genera, while *Syntrophorhabdus* showed the most positive correlation with BIO treatment (Fig. 9S). Here, we also analysed the relationships between soil chemical properties and bacteria communities by RDA (Fig. 5A). The first two coordinate axes showed 64.94% and 16.96% of the total variation (P > 0.05). The total N, Exchangeable K, and Extractable P were the significant effective factors. *Clostridium_ sensu_ stricto_12* was positively correlated with total N, but negatively correlated with the other factors. *Clostridium_ sensu_ stricto_1* was positively correlated with Exchangeable K, Hydrolytic N, total P and Organic Matter. *Haliangium* was positively correlated with the Extractable P, total K and pH. Furthermore, results from Pearson correlation analysis among yield, weed control efficiency, soil chemical properties and bacteria communities is presented in Fig. 5B. *Nonomuraea* and *Nitrospira* were significantly associated with yield. *Cercis_giantea* was negatively correlated with pH. *Pseudomonas* and *Sphingomonas* were negatively correlated with TotalK. *Candidatus_Nitosotalea* was associated with weed number control effect (NEF) and weed fresh weight control effect (WEF). Hence, soil bacterial communities were associated with soil chemical properties, weed control efficiency and yield under BIO-800 treatment.

## Discussion

Analysis of soil bacteria can provide important insights into their dynamic influences on farmland ecosystems and their “side-effect” biochemical processes (Pertile et al. 2021). In the present study, the alpha-diversity indices and PCA analysis of the different BIO treatments indicated that the soil bacterial diversity in the BIO-treated soil samples was not overall different from the untreated soil samples (Figs. 2 and 3). Taken together, the results demonstrated the recommended-dosage application of BIO did not have a significant influence on soil bacteria. Several previous papers have reported similar results to our work. For example, the application of imazethapyr (1.6 mg kg^− 1^) had no adverse effect on soybean soil microbial biomass and activity (Perucci and Scarponi 1994). Also, the methanotrophic community structure and prevalence did not differ between atrazine-treated and untreated soil (Seghers et al. 2003).

However, bacterial community structure was still changed between 2017 and 2021 after long-term application, especially that of the dominant genera. The genera *Anaeromyxobacter* and *Clostridium sensustricto1* were also the two most representative genera among the BIO-treated soil samples (Figs. 3 and 4S). The other three of the five dominant genera were *Candidatus Nitrosotalea*, *Haliangium*, and *Candidatus Nitrotoga* in 2017. However, in 2021 these changed to *SC-I-84*, *Muribaculaceae*, and *MBNT15*. The dominant population have a functional effect in the soil. For instance, *Muribaculaceae* is the longevity-linked microbiome in soil (Sibai et al. 2020). Several previous papers had reported similar results that organic fertilization had effect on soil bacterial communities. For example, results from a long-term field experiment on organic fertilization from 1989 to 2017 showed that the active bacterial diversity and composition did not show significant variations, but *Bacillus asahii* was the most striking differentially active bacteria (Su et al. 2021). Also, chitooligosaccharides was shown to enrich the abundance of *Clostridium sensustricto1*, which resulted in the production of fatty acids as main products (Ji et al. 2021). These fatty acids may also inhibit weeds, indicating that overall, the functional genus may become a dominant genus, which may be beneficial for weed suppression. Above that the change of soil bacterial communities were beneficial to weed management.

Meanwhile, the overusage of BIO obviously affected the bacterial community structure between 2017 and 2021. Similarly, the application of high concentrations of bio-organic fertilizer (BIO10 and BIO20) was reported to have significantly reduced disease incidence by 33.3-66.7% and manipulated the composition of soil microbial community (Huang et al. 2017). Also, a terminal restriction fragments length polymorphism analysis showed that soil fungal communities differed significantly between soil to which 40 g/kg seaweed fertilizer was applied and that to which 0, 5, and 20 g/kg was applied (Wang et al. 2016). The genera which showed significant difference between BIO-800 treated and untreated soils, were *Clostridium_sensu_stricto_1*, *Syntrophorhabdus*, *Candidatus_ Koribacter*, *Rhodanobacter*, *Bryobacter*, *Haliangium*, *Anaeromyxobacter* (Fig. 5A). As previously reported, *Rhodanobacter* was able to assimilate fatty acids, which was used to control weeds (Dahal & Kim 2017). Interestingly, *Syntrophorhabdus* fermented phenols into easily biodegradable substrates, which served as a keystone for soil ecosystem maintenance (Zheng et al. 2020). *Bryobacter* genera showed significantly correlations with amino acids and sugars acids (Liu et al. 2020). Anaeromyxobacter dehalogenans have a functional effect in arsenic release from these environments (Kudo et al. 2013). Hence, the overusage of BIO had a positive effect on weeds, but no obvious influence on bacterial community structure.

Soil bacterial communities play roles in soil chemical properties and enzymatic activity. RDA analysis showed that *Clostridium_ sensu_ stricto_12* was positively correlated with total N. *Clostridium_ sensu_ stricto_1* was positively correlated with Exchangeable K, Hydrolytic N, total P and Organic Matter (Fig. 5A). Previous studies have also suggested that *Candidatus Koribacter* have a beneficial effect on the yield of crop (Zhou et al. 2019). *Haliangium* is a sensitive genus which was shown to have negatively correlated with abiotic stress (Uddin et al. 2019). Extractable P and Exchangeable K were key environmental elements in bacterial community (Jiang et al. 2019). Meanwhile, S-ACP and S-UE activities were not influenced by BIO-treatment compared to CK in 2021 (Fig. 2B and C), but S-β-GC activity was obviously influenced under BIO-treatment (Fig. 2D). Similarly, it was reported that *Cyanobacteria* combined with *Arundo donax* played an important role in enhancing S-UE and S-ACP activity (Zeng et al. 2019). Therefore, the variations in soil bacterial communities may influence similar variations in the soil chemical properties and enzymatic activities.

Application of high dosages of BIO had effects on soil bacterial community, soil chemical properties and enzymatic activities. The genera *Anaeromyxobacter* and *Clostridium sensustricto1* were the two most representative of dominant genera among BIO-treated soil samples. *Clostridium_ sensu_stricto_1* also was the genus which showed significant difference between BIO-800 treated and untreated soils. BIO treatment may affect soil Exchangeable K, Hydrolytic N, and Organic Matter, thereby affecting bacterial communities, which in turn could affect weed control, and result in the yield improvement. This study indicated that the appropriate application of BIO will effectively manage weeds and yields in rice paddies, but will not have negative effects on soil microbial functions. However, some nutrient contents such as Extracted phosphorus and total K and N are the main factors which influenced bacterial community, due to over-enrichment of soil nutrition driven by the long-term overuse of BIO fertilizer. Further works are needed to explore the functional profiles of differentially-active soil bacteria under long-term BIO application.

## Electronic supplementary material

Below is the link to the electronic supplementary material.

Supplementary Material 1

## Data Availability

The 16sRNA sequence data that support the findings of this study are openly available in NCBI. The associated BioProject number is PRJNA872290. The associated SRA numbers are SRR13517891-SRR13517921, respectively.
