# Supplementary material for: Effect of long-term application of bioorganic fertilizer on the soil property and bacteria in rice paddy
Source: AMB Express. 2023 Jun 13;13:60. doi: 10.1186/s13568-023-01559-2 (PMC10264312; doi:10.1186/s13568-023-01559-2)
Supplement: Supplementary file 1 — Supplementary Material 1 [file 13568_2023_1559_MOESM1_ESM.docx]

***Supplementary materials* for**

**Effect of long-term application of bioorganic fertilizer on the soil property and bacteria in rice paddy**

LI Zu-ren ^*^, LUO Si-quan, Peng Yajun, JIN chen-zhong, LIU Du-cai^*^

Key laboratory of Pesticide Assessment, Ministry of Agriculture and Rural Affairs, P.R. China, Hunan Provincial Key Laboratory for Biology and Control of Weeds, Hunan Academy of Agricultural Sciences, Changsha 410125, China

^*^Corresponding authors. E-mail: [lizuren88214@hunaas.cn,](mailto:lizuren88214@hunaas.cn,) [ducailiu@163.com](mailto:ducailiu@163.com), Phone: 86-0731-84696075

**Table1S**.Chemical properties of the surface soil layer (0–15cm) from the BIO-treated and untreated sites in 2017.

| Variable source | pH | Total K  g/kg | Total N  g/kg | Total P  g/kg | Exchangeable K  mg/kg | ExtractableP  mg/kg | Hydrolytic  N  mg/kg | Organic matter  g/kg |
| --- | --- | --- | --- | --- | --- | --- | --- | --- |
| BIO-50 | 5.25 | 18.6 | 1.4 | 0.45 | 38 | 0.21 | 133.67 | 19.90 |
| BIO-100 | 5.17 | 18.53 | 1.44 | 0.50 | 45.67 | 0.36 | 147.67 | 21.63 |
| BIO-200 | 5.54 | 19.77 | 1.84 | 0.58 | 80.67 | 0.36 | 220 | 25.47 |
| BIO-400 | 5.36 | 19.1 | 1.53 | 0.46 | 76 | 0.42 | 177.67 | 21.57 |
| BIO-800 | 5.61 | 19.67 | 1.62 | 0.50 | 108.33 | 0.45 | 219.67 | 22.07 |
| CK | 5.36 | 18.63 | 1.51 | 0.49 | 56b | 0.22 | 165.33 | 21.97 |
| HPS | 5.22 | 18.90 | 1.48 | 0.50 | 42 | 0.25 | 142.67 | 20.67 |
| CBFS | 5.32 | 19.13 | 1.36 | 0.51 | 37.67 | 0.45 | 146.33 | 21.1 |

**Table2S**.Chemical properties of the surface soil layer (0–15cm) from the BIO-treated and untreated sites in 2021

| Variable source | pH | Total K  g/kg | Total N  g/kg | Total P  g/kg | Exchangeable K  mg/kg | ExtractableP  mg/kg | Hydrolytic  N  mg/kg | Organic matter  g/kg |
| --- | --- | --- | --- | --- | --- | --- | --- | --- |
| BIO-50 | 5.10 | 18.80 | 2.06 | 0.72 | 144.33 | 1.37 | 281.33 | 30.60 |
| BIO-100 | 5.17 | 18.87 | 2.00 | 0.71 | 174.67 | 2.77 | 298.33 | 30.67 |
| BIO-200 | 5.36 | 19.17 | 2.08 | 0.84 | 196.67 | 4.10 | 295.33 | 32.43 |
| BIO-400 | 5.36 | 19.37 | 2.01 | 0.84 | 212.00 | 7.10 | 315.00 | 31.90 |
| BIO-800 | 5.70 | 19.43 | 2.08 | 0.92 | 245.33 | 9.30 | 351.67 | 35.03 |
| CK | 5.09 | 18.53 | 1.92 | 0.57 | 91.67 | 1.07 | 217.67 | 26.83 |
| HPS | 5.12 | 18.90 | 1.94 | 0.71 | 91.33 | 2.00 | 217.00 | 27.53 |
| CBFS | 5.19 | 19.20 | 2.01 | 0.57 | 149.67 | 1.13 | 246.00 | 29.53 |


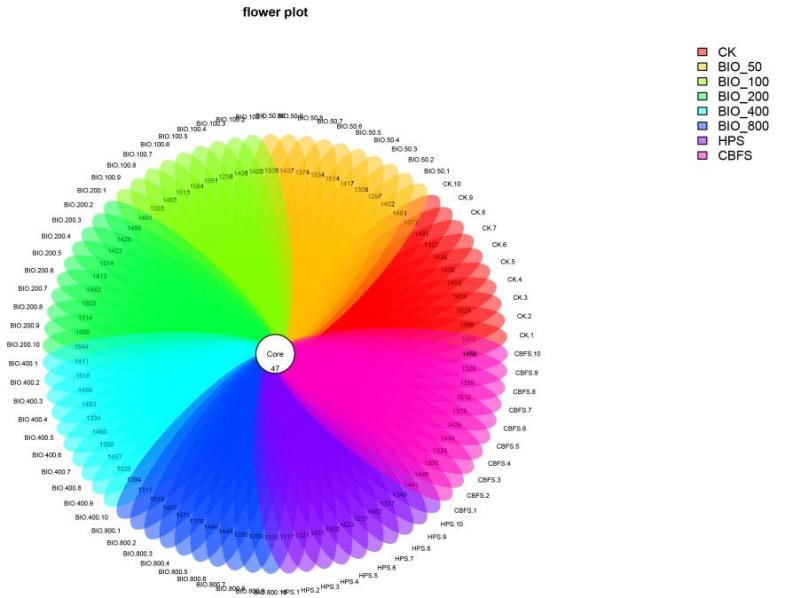


Fig.1S Operational taxonomic units (OTU) based petal map (flower plot) of soil samples in 2017. Description: Different colors in the diagram represent a (group) sample. Middle core numbers represent the number of OTUs common in all samples and numbers on the petals represent the number of OTU unique to this sample. Samples in the flower plots are as follows: BIO-treated soil samples, CBF soil samples, HP soil samples and CK. The acronyms denote the following: BIO_50: 750 kg/ha BIO-treated soil; BIO_100: 1500 kg/ha BIO-treated soil; BIO_200: 3000 kg/ha BIO-treated soil; BIO_400: 6000 kg/ha BIO-treated soil; BIO_800: 12000 kg/ha BIO-treated soil; HPS: herbicide-treated soil; CBFS: common bio-fertilizer-treated soil, and CK: untreated control soil.


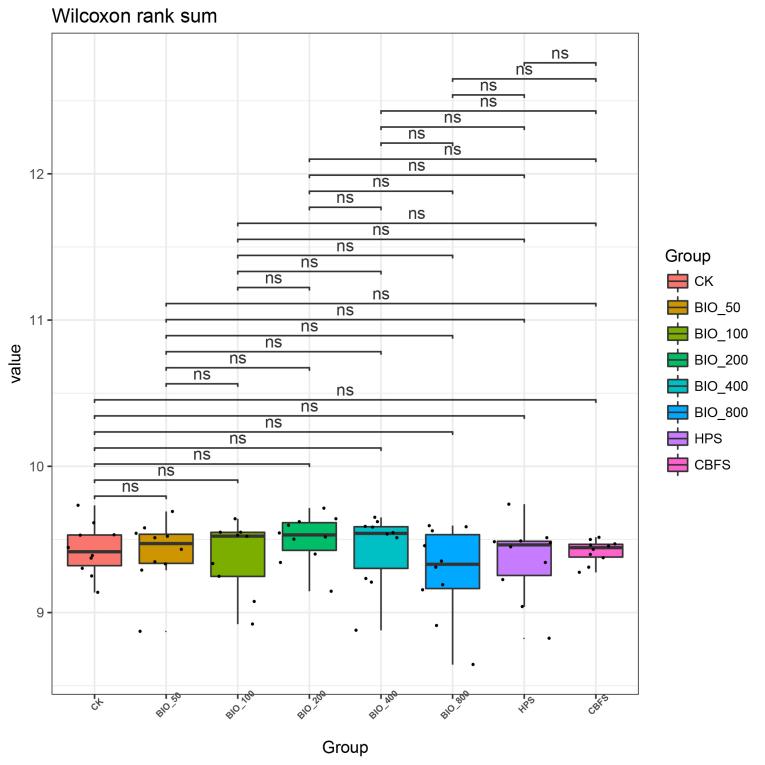


Fig.2S. Alpha-diversity indices, Shannon indices, of the bacterial community structure in BIO treatment; The acronyms denote the following: BIO_50: 750 kg/ha BIO-treated soil; BIO_100: 1500 kg/ha BIO-treated soil; BIO_200: 3000 kg/ha BIO-treated soil; BIO_400: 6000 kg/ha BIO-treated soil; BIO_800: 12000 kg/ha BIO-treated soil; HPS: herbicide-treated soil; CBFS: common bio-fertilizer-treated soil, and CK: untreated control soil. Data analysis is based on the average of nine repetitions. Means with the same letter are not significantly different according to Fisher’s protected LSD test (a = 0.05).


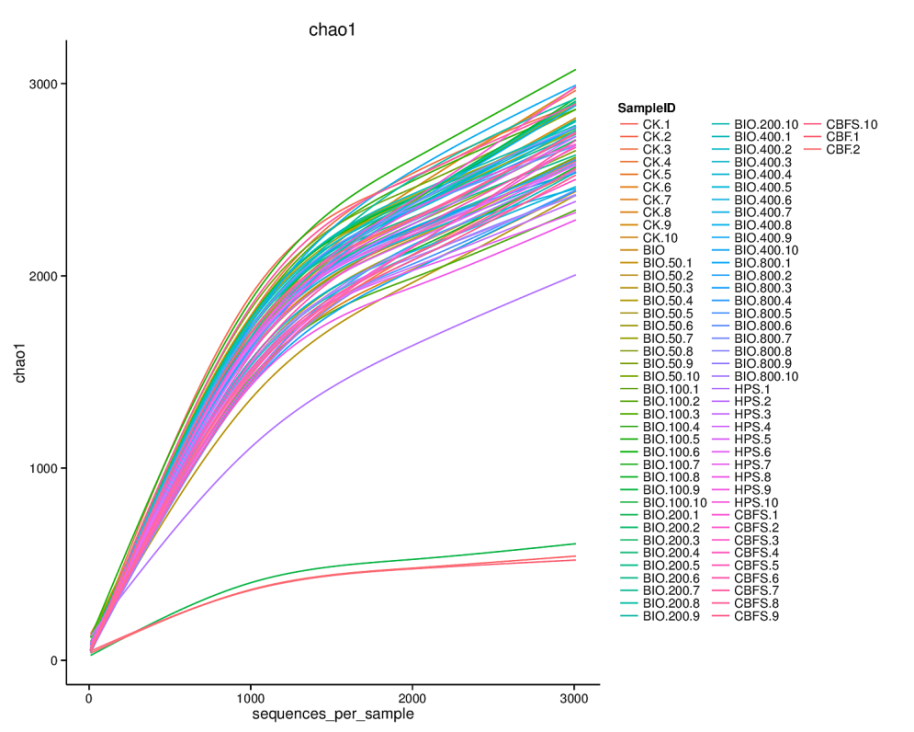


Fig.3S. Alpha-diversity indices, chao1 indices, of the bacterial community structure in BIO treatment; The acronyms denote the following: BIO_50: 750 kg/hm^2^ BIO-treated soil; BIO_100: 1500 kg/hm^2^ BIO-treated soil; BIO_200: 3000 kg/hm^2^ BIO-treated soil; BIO_400: 6000 kg/hm^2^ BIO-treated soil; BIO_800: 12000 kg/hm^2^ BIO-treated soil; HPS: herbicide-treated soil; CBFS: common bio-fertilizer- treated soil, and CK: untreated control soil.


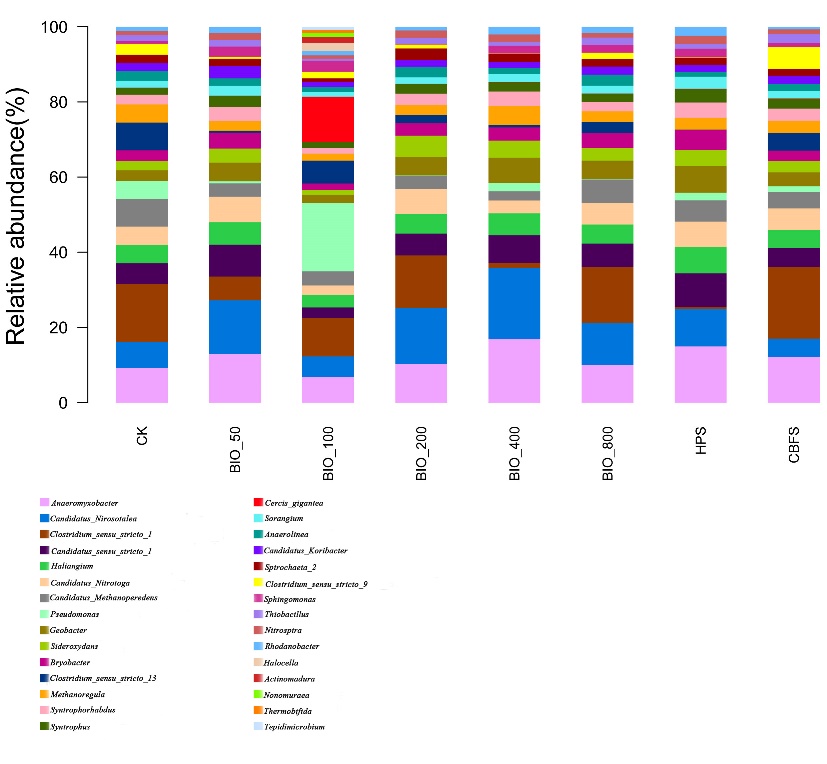


Fig.4S.Bacterial diversity, as represented by the relative abundances (%) of different genera in BIO-treated, CBF, and HP soil samples, and CBF and BIO samples, respectively. “others” refers to 16S sequence analysis that are not strictly associated with genus;

BIO: BIO sample; BIO-50: 750 kg/hm^2^ BIO-treated soil in 2017; BIO-100: 1500 kg/hm^2^ BIO-treated soil in 2017; BIO-200: 3000 kg/hm^2^ BIO-treated soil in 2017; BIO-400: 6000 kg/hm^2^ BIO-treated soil in 2017; BIO-800: 12000 kg/hm^2^ BIO-treated soil in 2017; HPS: herbicide-treated soil in 2017; CBF: common bio-fertilizer in 2017; CBFS: common bio-fertilizer-treated soil in 2017; and CK: untreated control soil in 2017.


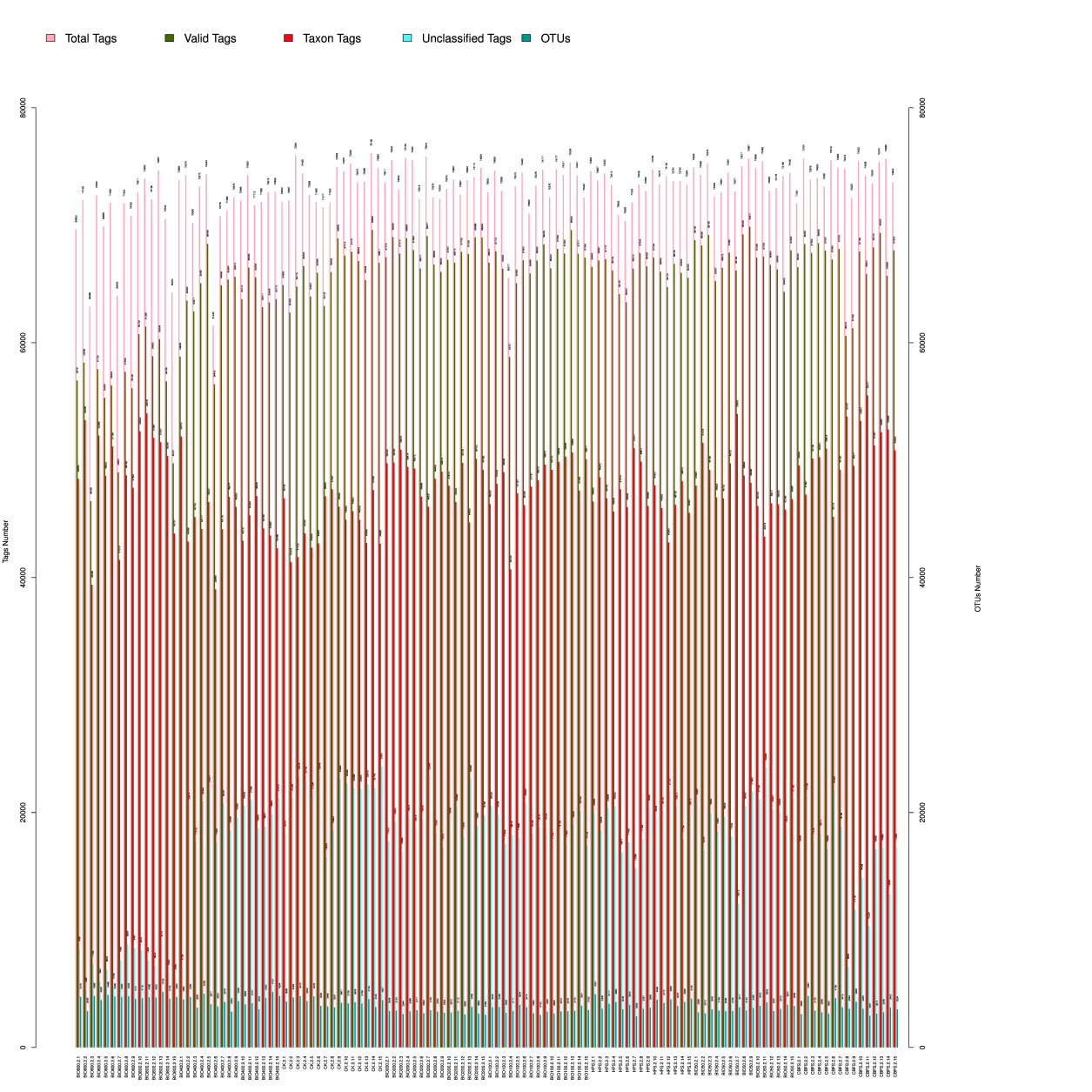


Fig.5S OTU annotation barplot of genus in all samples in 2021

Number 1 Y-axis showed the tags number in all samples, Number 2 Y-axis showed

OUTs of all samples. Total tags: Clean tags number of each sample; Valid tags: Clean tags number of each sample exclude chimera; Taxon tags: number of annotation valid tags; Unclassified tags: number of unannotation valid tags.


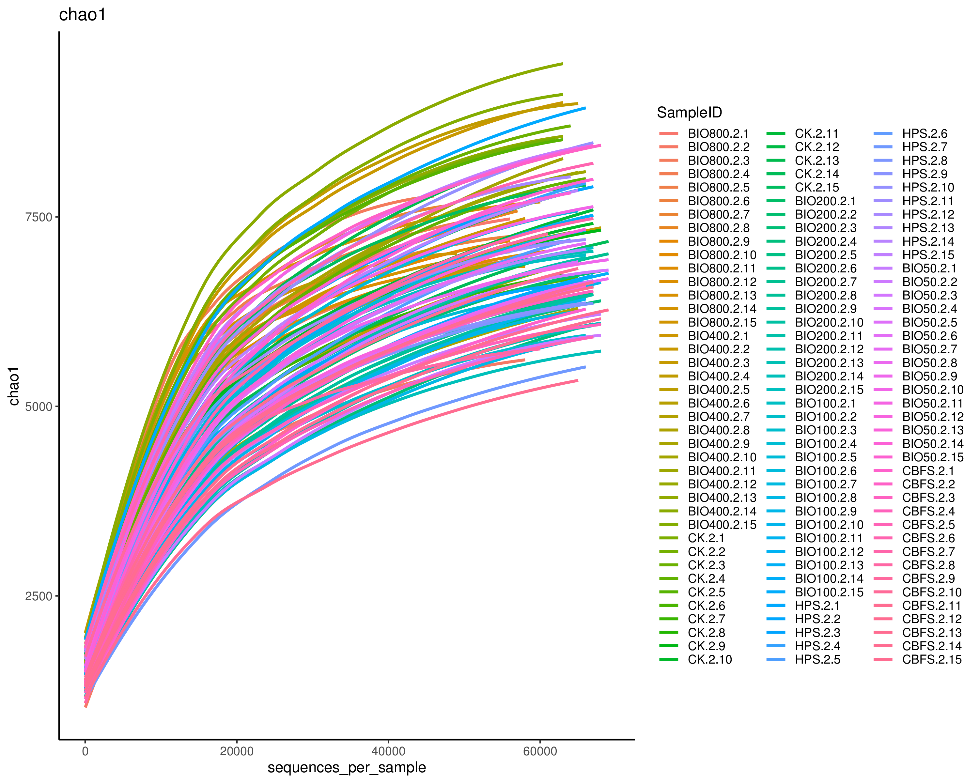


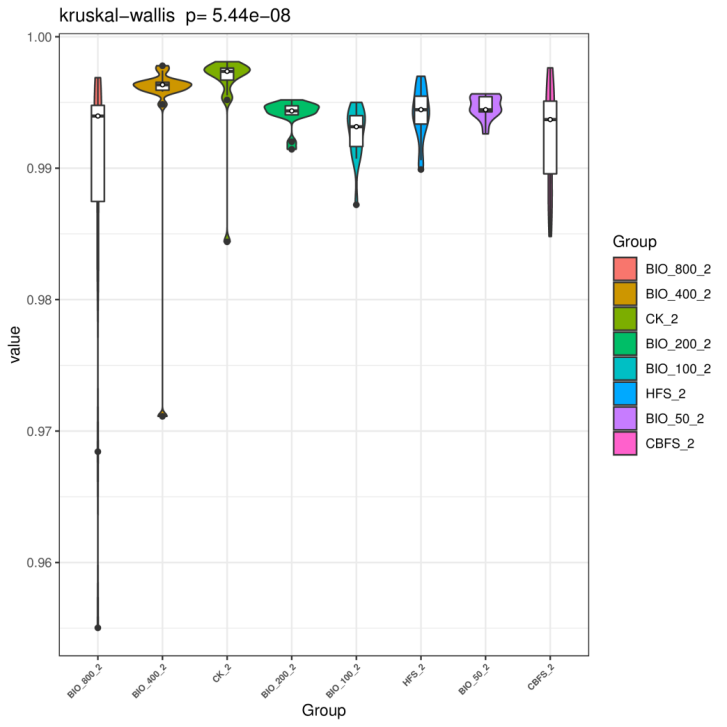
Fig.6S Alpha-diversity indices, chao1 indices of the bacterial community structure in the following samples: BIO application soil samples, CBF soil samples, HP soil samples, CBF samples, and BIO samples. The acronyms denote the following: BIO_2: BIO sample; BIO-50_2: 750 kg/ha BIO-treated soil; BIO-100_2: 1500 kg/ha BIO-treated soil;BIO-200_2: 3000 kg/ha BIO-treated soil; BIO-400_2: 6000 kg/ha BIO-treated soil; BIO-800_2: 12000 kg/ha BIO-treated soil; HPS_2: herbicide-treated soil; CBF_2: common bio-fertilizer; CBFS_2: common bio-fertilizer-treated soil, and CK_2: untreated control soil.

Fig.7S Alpha-diversity indices, simpson indices of the bacterial community structure in the following samples: BIO application soil samples, CBF soil samples, HP soil samples, CBF samples, and BIO samples. The acronyms denote the following: BIO_2: BIO sample; BIO-50_2: 750 kg/ha BIO-treated soil; BIO-100_2: 1500 kg/ha BIO-treated soil;BIO-200_2: 3000 kg/ha BIO-treated soil; BIO-400_2: 6000 kg/ha BIO-treated soil; BIO-800_2: 12000 kg/ha BIO-treated soil; HPS_2: herbicide-treated soil; CBF_2: common bio-fertilizer; CBFS_2: common bio-fertilizer-treated soil, and CK_2: untreated control soil.


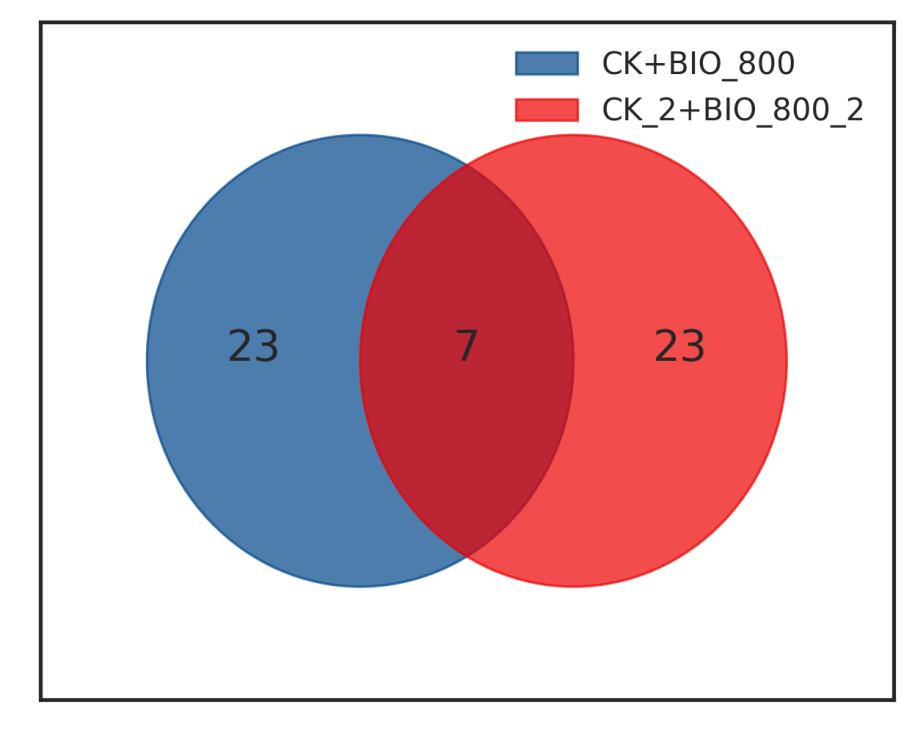


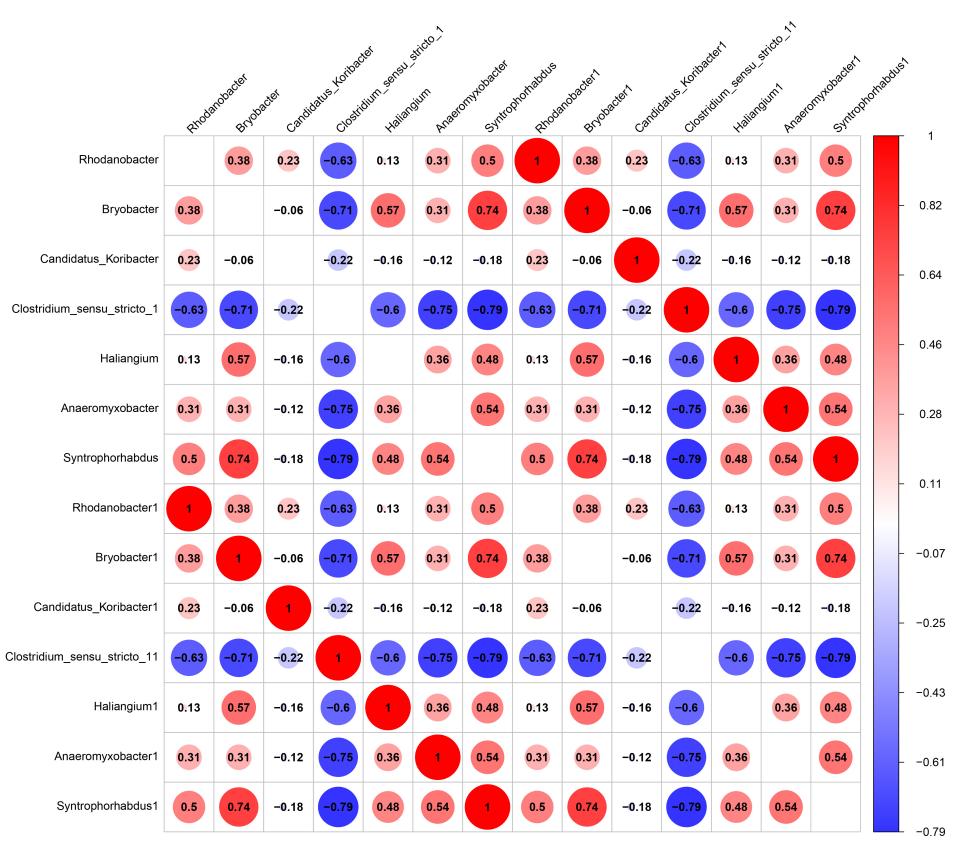
Fig.8S. Venn diagram of differentially active soil bacterial genera between BIO-800 and CK among 2017 and 2021; BIO-800: 12000 kg/ha BIO-treated soil in 2017; BIO-800_2: 12000 kg/ha BIO-treated soil in 2021;

Fig.9S. The networks visualize the influence by BIO-800 treatment on co-occurrence pattern between differentially active genera. A connection stands for a strong (Spearman's P>0.6) and significant (P<0.05) correlation. The size of each node is proportional to the number of connections (that is, degree).Positive correlations are colored red, while negative correlations are colored blue.

**Table3S. The abbreviation of the paper**

| Abbreviation | Full name |
| --- | --- |
| BIO | bioorganic fertilizer |
| BIO50 | 750 kg/hm^2^ BIO-treated soil in 2017 and 2021 |
| BIO100 | 1500 kg/hm^2^ BIO-treated soil in 2017 and 2021 |
| BIO200 | 3000 kg/hm^2^ BIO-treated soil in 2017 and 2021 |
| BIO400 | 6000 kg/hm^2^ BIO-treated soil in 2017 and 2021 |
| BIO800 | 12000 kg/hm^2^ BIO- treated soil in 2017 and 2021 |
| HPS | herbicide-treated soil in 2017 and 2021 |
| CBF | common bio-fertilizer in 2017 and 2021 |
| CBFS | common bio-fertilizer-treated soil in 2017 and 2021 |
| CK | untreated control soil in 2017 and 2021 |
| S-UE | Soil urease |
| S-ACP | Soil acid phosphatase |
| S-β-GC | Soil β-glucosidase |
| PCA | principal coordinates analysis |
| RDA | Redundancy analysis |
| OTU | Operational taxonomic unit |
